# Engineered Nanomaterial-Based Photocatalysts for Efficient and Sustainable Degradation of Antibiotics in Water: A Review

**DOI:** 10.1021/acsomega.6c00310

**Published:** 2026-04-06

**Authors:** Jyoti Rawat, Charu Dwivedi, Bertin Anzaldo, Pankaj Sharma

**Affiliations:** † 231512 Instituto de Química, UNAM, Circuito Exterior, Coyoacán CDMX 04510, México; ‡ Department of Chemistry, School of Physical Sciences, Doon University, Dehradun, Uttarakhand 248001, India; § 3972 Benemérita Universidad Autónoma de Puebla, Laboratorio de Síntesis de Complejos, Facultad de Ciencias Químicas, Puebla, Pue. C.P.72592, México

## Abstract

The widespread use of antibiotics in healthcare, agriculture, and animal husbandry has led to their persistent presence in aquatic environments, posing significant ecological and public health risks. Photocatalytic degradation has emerged as a promising advanced oxidation process for the efficient breakdown of antibiotics in water. Owing to their high surface area, directional charge transport, and enhanced reactive oxygen species generation, one-dimensional (1D) nanomaterials exhibit superior photocatalytic performance. Unlike previous reviews, this study integrates synthesis strategies, material characterization, degradation mechanisms, and evaluations of stability and reusability, while systematically addressing reproducibility, scalability, and long-term operational stability, key challenges that determine the reliability and practical deployment of photocatalytic systems. Particular attention is given to synthesis consistency, performance variation across repeated cycles, and resistance to photocorrosion and material degradation under realistic operating conditions. These considerations provide mechanistic insights and help identify future research directions. The discussion highlights the environmental impact of antibiotic contamination, the principles of photocatalytic degradation, and recent advances in nanostructured photocatalytic materials for sustainable wastewater treatment. By emphasizing the unique properties, reproducibility, and durability of 1D nanomaterials, this review presents a distinct and focused perspective on addressing persistent antibiotic pollutants in water.

## Graphical abstract

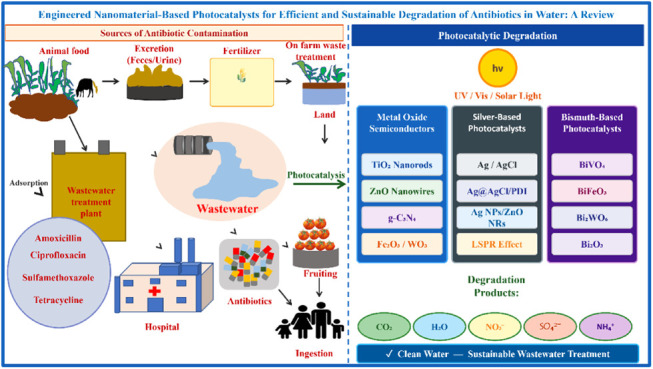

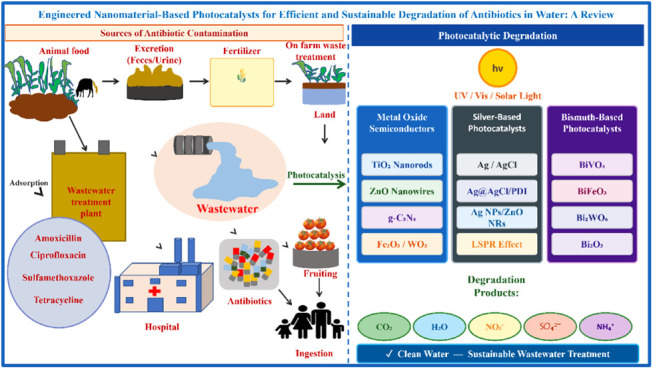

## 1 Introduction

Antibiotics, a cornerstone of modern medicine, have been extensively used to treat bacterial infections in humans, animals, and agricultural practices.^1^ However, their widespread and often indiscriminate use has promoted the emergence and proliferation antibiotic-resistant bacteria, leading to their persistent accumulation in the environment. A substantial portion of consumed antibiotics are excreted unmetabolized and enter wastewater systems, eventually reaching surface waters and even drinking water supplies.^2,3^ Traditional wastewater treatment plants (WWTPs) are generally ineffective at removing these biologically active micropollutants, resulting in long-term environmental exposure.^4^ Even at low concentrations, antibiotics can disrupt natural microbial communities, alter nutrient cycling, and promote the dissemination of antibiotic resistance genes (ARGs).^5^ Therefore, developing advanced, efficient, and sustainable technologies for antibiotic removal has become an urgent priority. Among these, photocatalytic degradation has emerged as a sustainable and highly effective method for breaking down recalcitrant organic pollutants such as antibiotics.

In recent years, nanotechnology has shown considerable promise in environmental remediation, particularly in water purification and contaminant degradation.^5^ 1D nanostructures such as nanowires (NWs), nanorods (NRs), and nanotubes (NTs) have attracted interest because of their distinctive structural and functional characteristics.^4,5^ These materials exhibit high aspect ratios, and superior electron transport characteristics, making them especially suitable for photocatalytic applications.^6^ The efficiency of photocatalysis depends heavily on the photocatalyst’s properties, including surface area, band gap, and separate charge carriers.^7^ The elongated geometry of 1D nanostructures promotes directional charge transport, and limits the recombination of photoexcited electrons and holes, thereby improving the overall photocatalytic efficiency.^5,6^ However, not all 1D structures perform equally under the same conditions. For example, TiO_2_ NRs often show higher photocatalytic activity than ZnO NWs under UV light due to their more favorable band structure and surface defect density, whereas carbon nanotubes (CNTs) excel in visible-light applications because of their excellent conductivity.^7^ This indicates that the choice of material and light source critically determines performance, and that morphology alone does not guarantee superior activity.^8^ Additionally, studies report contradictions regarding degradation efficiency, some ZnO NWs outperform TiO_2_ NRs in certain pollutant systems, likely due to differences in surface functionalization or experimental conditions, highlighting the need for standardized testing.^9^

From an engineering feasibility and scalability perspective, the practical deployment of 1D nanostructures in real water treatment systems requires. Catalyst recovery remains a significant challenge, particularly in slurry-based reactors where dispersed NWs or NTs must be separated after treatment, increasing operational cost and complexity.^10^ Immobilization approaches such as growing 1D nanostructures directly on glass, or metal substrates, incorporating them into membrane matrices, or fabricating aligned arrays on conductive supports can enhance recyclability and reduce the risk of secondary contamination.^11^ In continuous-flow or fixed-bed photoreactors, vertically aligned 1D architectures may improve light penetration, and facilitate directional mass and electron transport compared to randomly aggregated conventional nanoparticles. Nevertheless, large-scale synthesis, structural uniformity, mechanical robustness, and long-term stability under irradiation remain critical challenges.^11^ Although 1D nanostructures offer intrinsic advantages in charge transport and potentially easier recovery when immobilized, conventional nanoparticles often remain more cost-effective and simpler to manufacture.^12^ Therefore, at the system level, the real advantage of 1D nanostructures depends on successful integration into stable, and scalable reactor designs rather than morphology alone guaranteeing superior practical performance.^12^

Upon activation by light, typically in the ultraviolet (UV) or visible range, these materials produce reactive oxygen species (ROS), including hydroxyl radicals (•OH) and superoxide anions (O_2_•^–^).^7^ Integrating 1D nanostructures into photocatalytic systems significantly improves the degradation efficiency of antibiotics in water. Materials such as TiO_2_ NRs, ZnO NWs, and CNTs have demonstrated superior photocatalytic activity compared to their bulk or nanoparticulate counterparts.^13^ Despite these advantages, common limitations persist, potential aggregation of 1D structures, and challenges in large-scale synthesis, which must be addressed for practical applications.^14^ Beyond performance enhancement, this study contributes to environmental sustainability by promoting the use of green materials and eco-friendly substrates. These materials offer advantages such as natural abundance, and recyclability, making them ideal platforms for sustainable technologies. Their use enables the development of flexible, low-impact devices while reducing material waste and supporting environmentally responsible innovation.^14^

Numerous studies have reported successful degradation of antibiotics, including amoxicillin (AMX), sulfamonomethoxine (SMM), tetracycline (TC), ciprofloxacin (CIP), sulfadimethoxine (SDM) and sulfamethoxazole (SMZ or SDX), using 1D nanostructured photocatalysts.^15^ These antibiotics, due to their complex molecular structures and resistance to conventional treatment, are classified as persistent organic pollutants (POPs) that require advanced removal strategies. 1D nanomaterials have shown exceptional photocatalytic performance under both UV and visible light, with their unique morphological and electronic properties high surface-to-volume ratios, directional charge transport pathways, and increased active site density contributing to superior degradation efficiencies compared to bulk materials.^15,16^

Despite these advancements, a variety of approaches have been explored over the past decade to remove antibiotics from wastewater, underscoring the ongoing challenge of effective elimination.^9^ Conventional methods include optimized biological treatment and membrane-based technologies, while advanced approaches encompass advanced oxidation processes, adsorption using activated carbon or biochar, and constructed wetlands. However, the complex chemical structures and recalcitrant nature of antibiotics continue to limit treatment efficiency, highlighting the need for integrated and sustainable solutions.^11^ Several studies have explored specific removal strategies. Yang et al. investigated the fate of sulfonamide antibiotics (SMX, SMM, and SDM) in activated sludge with and without NaN_3_. Removal occurred via both adsorption and biodegradation, although biodegradation was initially inhibited. Adsorption was weak at neutral pH and followed a linear Freundlich isotherm, with affinity in the order SDM > SMM > SMX, and most adsorbed antibiotics were reversibly desorbed.^17^ Similarly, Liu et al. examined the removal of trace antibiotics from WWTP effluent using nanofiltration followed by advanced oxidation of the resulting concentrate. While UV treatment alone was ineffective, ozone-based processes, particularly the UV/O_3_ system, efficiently degraded antibiotics via hydroxyl radical generation. Treatment of real NF concentrate not only removed antibiotics effectively but also reduced toxicity and enhanced biodegradability, demonstrating the potential of combined NF-UV/O_3_ approaches.^18^ Sanga et al. prepared BiFeO_3_/MXene nanocomposites to remove six sulfonamide antibiotics from water. The BiFeO_3_ enhanced surface area and adsorption efficiency, outperforming pure MXene. The material showed pseudo-second-order kinetics, Langmuir adsorption behavior, good stability, and reusability over five cycles, providing an effective approach for sulfonamide removal.^19^ Chen et al. synthesized Ag@AgCl/PDI photocatalyst that efficiently degrades pollutants, and resistance genes under visible light due to improved light absorption and charge separation.^20^ Zisti et al. developed a magnetic CoFe_2_O_4_@3D-TiO_2_/graphene aerogel for visible-light degradation of AMX. It achieved complete removal within 120 min under optimal conditions, showed high reusability, and converted pollutants into nontoxic products. ROS drove the process, and the material’s magnetism allowed easy recovery, highlighting its potential for wastewater treatment.^21^ Zhang et al. prepared Cu-loaded porous geopolymer (Cu-PG) for persulfate-driven degradation of SMX, achieving 91.9% removal at pH 11 in 90 min. The process involved radical and nonradical pathways, with Cu­(III) as a key oxidant, and 6 degradation routes were proposed.^22^ Chen et al. developed a MoS_2_/BaSO_4_/zeolite (MBZ) composite that efficiently activates peroxymonosulfate under visible light to degrade SDZ. The hybrid enhanced charge separation and transfer, achieving higher removal rates than the individual components, providing a promising strategy for wastewater remediation.^23^

Recent studies have increasingly emphasized the role of sustainable and green materials in antibiotic monitoring and remediation. Martins highlights sustainable materials, including biomass-based platforms, for antibiotic detection and removal, with nanotechnology and AI enabling efficient, multifunctional solutions for antibiotic pollution.^24^ Ozório et al. reported a paper-based SERS platform using silver nanoparticle-decorated zinc oxide NRs. The ZnO NRs/AgNPs substrates achieved improved detection performance and stability for rhodamine 6G and thiabendazole compared with AgNP-only substrates, highlighting their suitability for low-cost environmental monitoring.^25^

Building on these findings, this review on the development and application of advanced 1D nanostructured photocatalysts for efficient antibiotic removal from water. This study aims to systematically analyze the synthesis and characterization of novel 1D nanomaterials or nanocomposites with enhanced light-harvesting capabilities, and excellent photocatalytic activity. Their efficiency in degrading selected antibiotic pollutants under both UV and visible light will be evaluated. By investigating degradation mechanisms, and reusability, this work seeks to advance sustainable, high-efficiency photocatalytic strategies for wastewater treatment and environmental protection.

## 2 Methods for Antibiotic Degradation

### 2.1 Classification of Antibiotic Degradation

Antibiotics are compounds that may be naturally derived, chemically synthesized, or semisynthetic substances that interfere with the growth or metabolic functions of microorganisms.^26^ Antibiotic degradation can be broadly classified into four main groups: chemical degradation, oxidative degradation, enzymatic degradation, and photodegradation. Chemical degradation refers to the spontaneous breakdown of antibiotic molecules under various physicochemical conditions, such as moisture, or oxidative environments. Hydrolysis is one of the most prevalent forms of chemical degradation, particularly affecting β-lactam antibiotics such as penicillin and cephalosporins.^27^ These compounds are highly sensitive to hydrolytic cleavage of the β-lactam ring, especially under acidic or basic conditions. Oxidative degradation is another pathway that affects antibiotics like tetracyclines and macrolides, resulting in the loss of biological activity due to structural modifications. Enzymatic degradation plays a prominent role in clinical antibiotic resistance. Certain bacteria possess enzymes that are capable of deactivating antibiotics.^28^ These enzymes work by breaking down the antibiotic molecules before they can reach their target sites within the bacterial cell.^28^ Other modifying enzymes, such as acetyltransferases, phosphotransferases, and nucleotidyltransferases, inactivate aminoglycosides through covalent modifications, impeding their ability to bind bacterial ribosomes.^28^ Photodegradation involves the light-induced breakdown of antibiotic compounds, particularly when exposed to UV radiation.^28^ This process is significant during drug manufacturing, packaging, and environmental exposure. Classes such as chloramphenicol, macrolides, trimethoprim, polypeptides, sulfonamides, mitomycins, and tetracyclines are especially susceptible to photodegradation, leading to the formation of inactive or even toxic degradation products.^26^ Therefore, light protection is crucial in the storage and formulation of these antibiotics (Table 1). Some antibiotics, such as penicillin, degrade easily, whereas others like tetracycline and tylosin are highly stable. These persistent antibiotics remain in the environment for extended periods, accumulate at high concentrations, and cause contamination. Although techniques such as photocatalysis, adsorption, and UV treatment have been used for antibiotic removal, their application is limited by low efficiency, and secondary chemical pollution.^29^

**Table 1:**
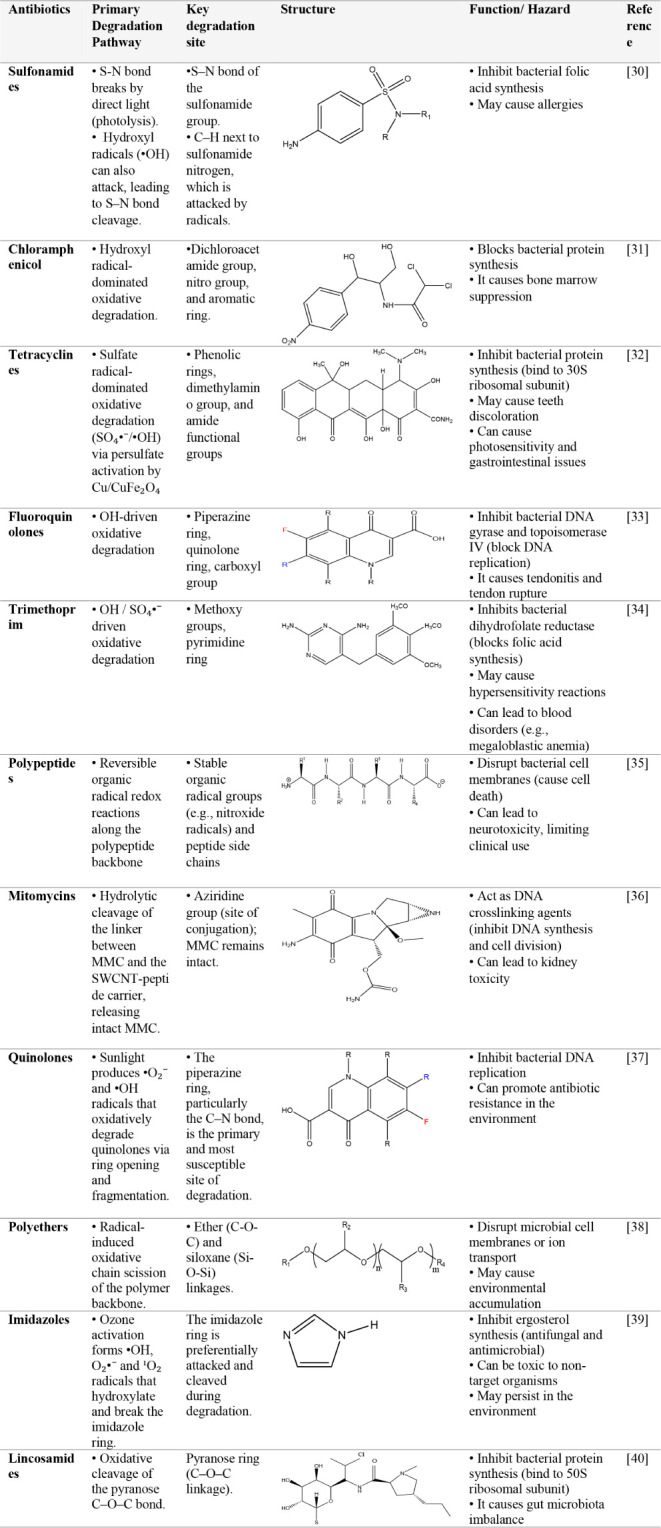
Classification of Different Antibiotics^30–40^

### 2.2 Photocatalytic Removal of Organic Pollutants

Photocatalysis is a light-induced process in which semiconductor materials absorb photons and generate electron–hole pairs that drive oxidation and reduction reactions on the catalyst surface.^41^ H^+^ and e^–^ react with water and oxygen to generate highly reactive species such as •OH and •O_2_^–^ radicals, which effectively degrade organic pollutants into harmless products like CO_2_ and H_2_O. Due to its strong oxidation ability, and environmental friendliness, photocatalysis is considered an efficient method for wastewater treatment.^42^ Consequently, it provides a sustainable and practical approach for the effective removal of contaminants from water.^43^ Ullah and Dutta investigated the photodegradation performance of Mn-doped ZnO nanoparticles by monitoring the breakdown of aniline and methylene blue (MB) dyes under visible light from a tungsten lamp. Their results indicated that Mn-doped ZnO exhibited superior photocatalytic activity compared to undoped ZnO.^44^ Yiming et al. synthesized GdVO_4_/g-C_3_N_4_ composites, which showed enhanced Rhodamine B (Rh–B) degradation due to improved charge separation at the heterojunction, with the 10 wt.% composite degrading dye over 3 times faster than pure g-C_3_N_4_.^45^ Haiqing et al. developed a SnIn_4_S_8_/Bi_2_MoO_6_ (BMOSIS) composite that efficiently generates H_2_O_2_ and degrades methyl orange (MO) under solar light, with the optimized BMOSIS_2_ showing the highest activity due to improved charge separation, and light absorption.^46^ Zubair et al. prepared two cobalt–carbon composites from MOFs, with N-doped Co-NCC showing higher porosity and superior catalytic activity for p-nitrophenol reduction than Co-CC, due to nitrogen sites and larger surface area.^47^ Jassal et al. demonstrated that nanocubes achieved photocatalytic degradation efficiencies of 94.15% for Malachite Green (MG) and 76.13% for Eriochrome Black T (EBT).^48^ Adekunle et al. reported that microwave-synthesized Fe_2_O_3_ nanoparticles achieved the highest degradation of Murexide (98%) and EBT (96%) at 25 mg catalyst over 40 min, while chemically synthesized Fe_2_O_3_ showed lower degradation, with Murexide at 15.2% and EBT at 21.4%.^49^

The degradation mechanism of EBT and Murexide is shown in eqs 1–4 below.

1 $${\mathrm{Fe}}_{2}O_{2}+h\nu \to {\mathrm{Fe}}_{2}O_{3}\left(e^{-}{+h}^{+}\right)$$

2 $$h^{+}(\mathrm{VB})+H_{2}O\to \mathrm{OH}•$$

3 $$e^{-}(\mathrm{CB})+O_{2}\to O_{2}^{-}$$

4 $${\mathrm{EBT}+\mathrm{Murexide}+h}^{+}{+O}_{2}^{-}+\mathrm{OH}\to \mathrm{degradation products}$$

## 3 Semiconducting Metal Oxide-Based Photocatalysts for the Degradation of Antibiotics

### 3.1 Metal Oxide Semiconductors as Photocatalysts

Semiconducting metal oxides play a pivotal role in photocatalysis because of their strong chemical stability, and favorable electronic characteristics that support efficient charge generation and transport.^50^ These materials can absorb light energy, leading to the formation of electron–hole pairs that subsequently drive surface redox reactions. The photocatalytic behavior of metal oxides is closely linked to their band gap, crystallinity, morphology, and surface characteristics. Wide-bandgap oxides such as TiO_2_ and ZnO have long been considered benchmark photocatalysts, primarily because of their strong oxidative potential, and high availability. However, their activity is largely limited to the UV region due to their band gaps (3.0–3.2 eV), which restricts their utilization under solar irradiation (Figure 1).^51^ Due to the wide band gaps of all TiO_2_ polymorphs, light absorption is largely confined to the UV region, limiting photocatalytic efficiency under solar irradiation. Additional challenges include rapid electron–hole recombination and weak interaction with hydrophobic organic pollutants.^51^ Matias et al. developed Ca-doped TiO_2_ integrated into cellulose membranes using a microwave-assisted method. Calcium doping created oxygen vacancies and structural defects, enhancing surface area and charge transport. The 10 mol.% Ca:TiO_2_ membrane achieved 81% tetracycline removal under solar light.^52^ Landolsi et al. fabricated a porous Fe_2_O_3_/TiO_2_ heterojunction through a simple two-step sol–gel and electrodeposition method. Under visible-light irradiation, the photocatalyst achieved approximately 70% MB degradation within 150 min, demonstrating superior performance compared to the individual oxides.^53^

**Figure 1:**
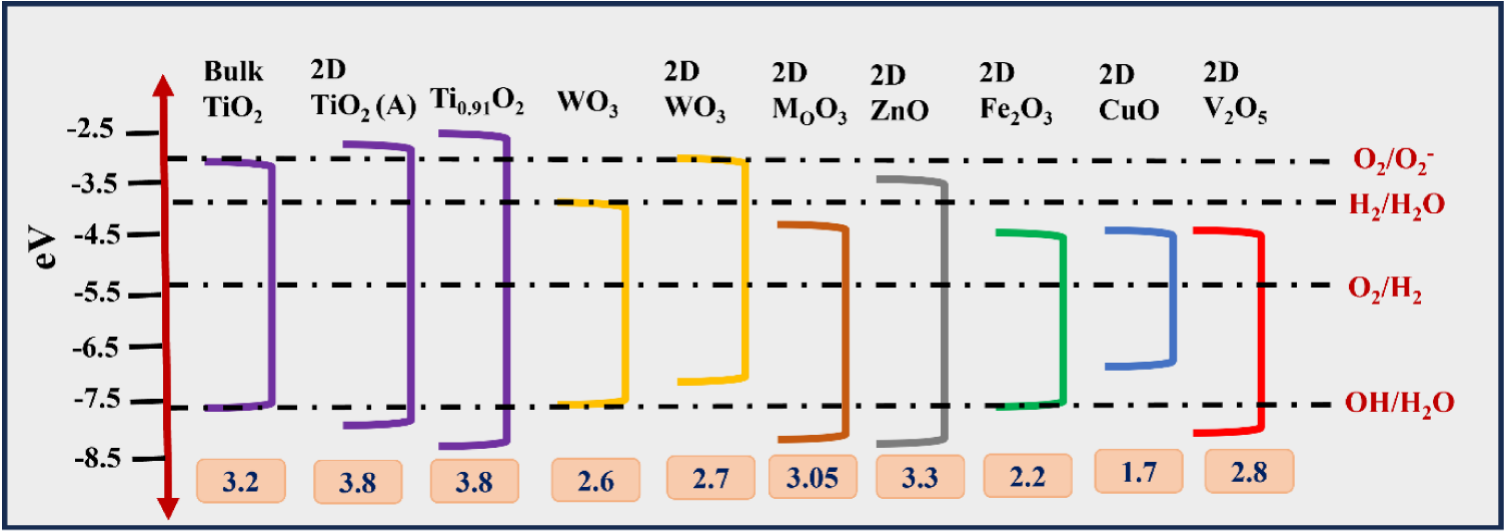
Energy band gaps of selected bulk semiconductors, showing the relative band edge positions with respect to redox potentials for water splitting.

In contrast, tungsten trioxide (WO_3_) has a band gap of 2.7 eV, allowing it to absorb a portion of the visible spectrum, although it suffers from fast electron–hole recombination. Molybdenum trioxide (MoO_3_), with a band gap of 3.05 eV, also exhibits modest visible light activity but is often limited by its low conduction band potential, affecting its efficiency in reduction reactions.^54^ Iron­(III) oxide (Fe_2_O_3_), or hematite, has a band gap of about 2.2 eV, enabling good visible light absorption, but its poor conductivity and short carrier diffusion length hamper overall photocatalytic performance.^55^ Copper­(II) oxide (CuO), a p-type semiconductor with a band gap of 1.7 eV, offers excellent absorption in the visible and near-infrared regions, yet its instability and high recombination rates remain challenges.^56^ Vanadium pentoxide (V_2_O_5_), with a band gap of 2.8 eV, is also active under visible light and shows promise in oxidation reactions, though its photocatalytic efficiency can be limited by structural instability under irradiation (Figure 1).^57,58^

Fabrication of composites or nanocomposites, such as nitrogen, sulfur, or carbon doping into TiO_2_, has been shown to introduce localized states near the VB, thereby reducing the effective band gap and extending photocatalytic activity into the visible light region. Transition metals like Fe, Cr, Mn, or Co, when doped into metal oxides, can serve as electron or hole traps, prolonging the lifetime of charge carriers and enhancing photocatalytic efficiency.^59^ However, excessive doping can introduce deep-level trap states that act as recombination centers, thus degrading performance. Therefore, optimizing the dopant concentration and distribution is critical to achieving the desired balance between enhanced light absorption and minimized recombination.^57^ Another key approach to enhance photocatalytic performance is through the construction of heterojunctions and composite systems. By combining two or more semiconductors with staggered band alignments, heterojunctions facilitate directional charge transfer, which spatially separates photogenerated electrons and holes and thereby reduces their recombination probability.^60^ These configurations mimic natural photosynthesis by using a mediator or direct interface to shuttle electrons, resulting in enhanced performance under solar light. Additionally, coupling metal oxides with carbonaceous materials like graphene, CNTs, or g-C_3_N_4_ has shown great promise in improving electrical conductivity and facilitating interfacial charge transfer, due to the favorable charge transport characteristics and high surface availability of these carbon materials.^61,62^

Applications of semiconducting metal oxide photocatalysts span a broad range, including water splitting for hydrogen generation, CO_2_ photoreduction to fuels, and the decomposition of environmental pollutants.^63,64^ For water splitting, the conduction and VB must straddle the hydrogen and oxygen redox potentials, respectively, and the material must remain chemically stable under both oxidative and reductive conditions. For CO_2_ reduction, adsorption and activation of CO_2_ on the surface is a major challenge, requiring the design of surface terminations or cocatalysts that facilitate selective multielectron transfer processes.^65,66^ Similarly, for environmental remediation, photocatalysts must maintain activity in the presence of complex aqueous matrices and resist fouling or deactivation by intermediate byproducts. In all these cases, the interplay between bulk charge transport, surface chemistry, and light absorption efficiency dictates the overall photocatalytic performance.^67^

### 3.2 Silver-Based Photocatalysts

Silver-based photocatalysts represent a versatile and strategically significant class of materials in the domain of solar-driven catalysis, particularly due to their unique high electrical conductivity, and tunable electronic structures.^68^ The inclusion of silver either as a metallic cocatalyst or as a primary photoactive component has been shown to markedly enhance the efficiency of photocatalytic processes through mechanisms such as localized surface plasmon resonance (LSPR), Schottky junction formation, and improved interfacial charge dynamics.^69^ One of the most compelling attributes of metallic silver (Ag^0^) is its ability to support LSPR, a phenomenon in which collective oscillations of conduction electrons are excited upon interaction with incident visible light. This effect not only broadens the spectral response of the photocatalyst but also enables the generation of energetic electrons capable of overcoming interfacial energy barriers and injecting into the conduction bands of adjacent semiconductors. Such plasmon-induced charge transfer substantially enhances photocatalytic activity by promoting carrier separation and suppressing recombination.^70^ Beyond its plasmonic behavior, silver also serves as an efficient electron acceptor due to its high work function, forming Schottky barriers at semiconductor interfaces. This facilitates the unidirectional migration of photogenerated electrons from the semiconductor to the Ag surface, where reduction reactions can occur more efficiently. The dual role of silver as both a plasmonic sensitizer and a cocatalyst distinguishes it from other noble metals traditionally used in photocatalysis.^71^

In addition to metallic silver, silver-containing compounds such as Ag_2_O, Ag_3_PO_4_, and silver halides (AgCl, AgBr, AgI) exhibit intrinsic photoactivity under visible light irradiation (Figure 2). For instance, Ag_3_PO_4_ possesses a direct bandgap of 2.4 eV and exhibits remarkable quantum efficiencies for oxygen evolution; however, its photocorrosion under prolonged illumination due to the reduction of Ag^+^ to metallic Ag remains a critical challenge.^72^ Hybrid architectures, such as Ag/AgX composites or Ag-semiconductor Z-scheme systems, have been developed to mitigate stability issues while leveraging synergistic charge transfer pathways.^73^ Despite their promising performance, silver-based photocatalysts face several limitations, including photocorrosion, agglomeration of nanoparticles, and potential environmental toxicity associated with silver ion leaching. To address these challenges, current research is increasingly focused on engineering advanced heterojunctions, optimizing particle morphology, and incorporating stabilizing matrices such as graphene, and CNTs.^74^ Moreover, in situ spectroscopic analyses and time-resolved spectroscopies are being employed to unravel the ultrafast dynamics of plasmon-induced processes and charge migration at the nanoscale.^75^

**Figure 2:**
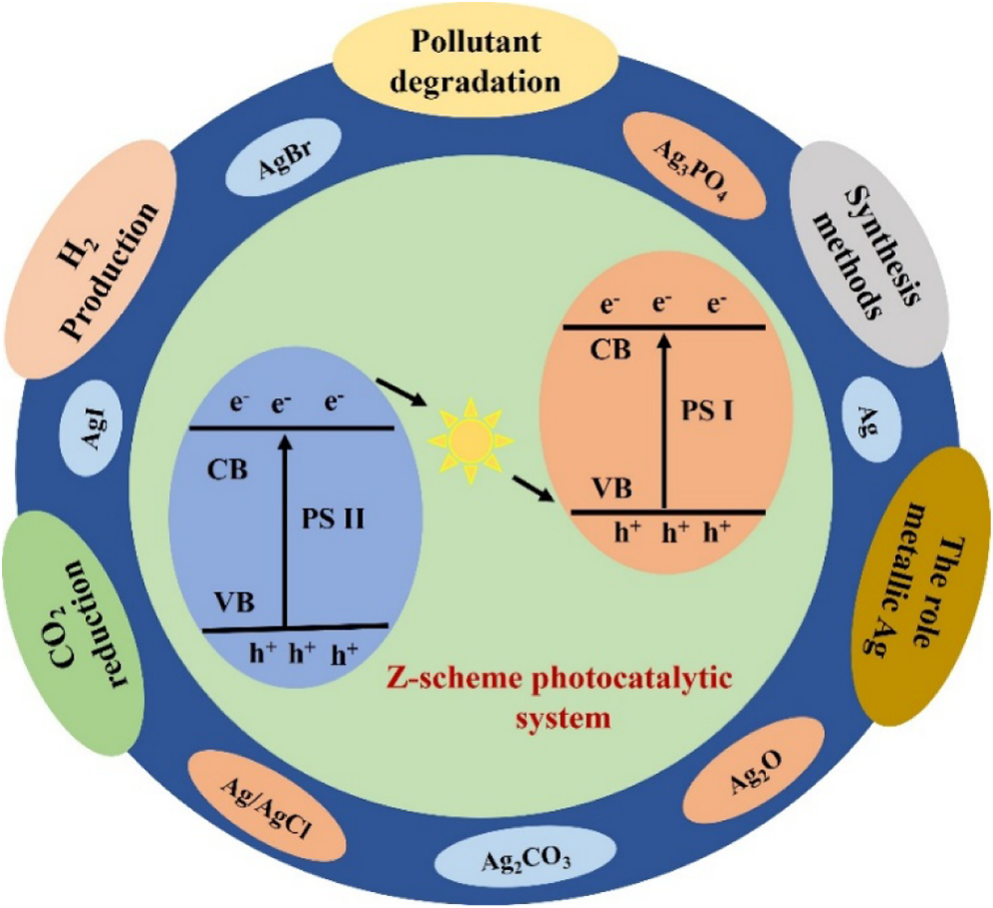
Schematic representation of the Z-scheme photocatalytic mechanism of silver-based materials. Upon light irradiation, electrons (e^–^) and holes (h^+^) are generated and transferred along the Z-scheme, producing reactive species responsible for pollutant degradation.

### 3.3 Bismuth-Based Photocatalysts

Bismuth-based photocatalysts are attractive for light-driven applications due to their tunable band gaps and strong visible light absorption. Unlike conventional wide-bandgap semiconductors such as TiO_2_ and ZnO, which require UV light for activation, many bismuth-containing compounds possess narrower bandgaps, enabling them to utilize visible light more effectively a critical advantage for environmental remediation.^76^ Structurally, bismuth-based materials often adopt layered, which introduce intrinsic anisotropic electric fields that facilitate the spatial separation of photogenerated electron–hole pairs. This behavior is particularly evident in compounds such as Bi_2_WO_6_, Bi_2_S_3_, BiOBr, BiOI, and Bi_2_WO_6_, whose internal polarization enhances charge carrier dynamics, thereby suppressing recombination losses and improving photocatalytic efficiency (Figure 3).^77^ Among these materials, monoclinic scheelite-phase BiVO_4_ has received substantial attention for water oxidation due to its suitable band alignment, visible-light response, and relative photochemical stability. Similarly, Bi_2_O_3_, with its polymorphic phases and high refractive index, offers diverse opportunities for photocatalytic design, although stability under prolonged irradiation remains a concern.^77^

**Figure 3:**
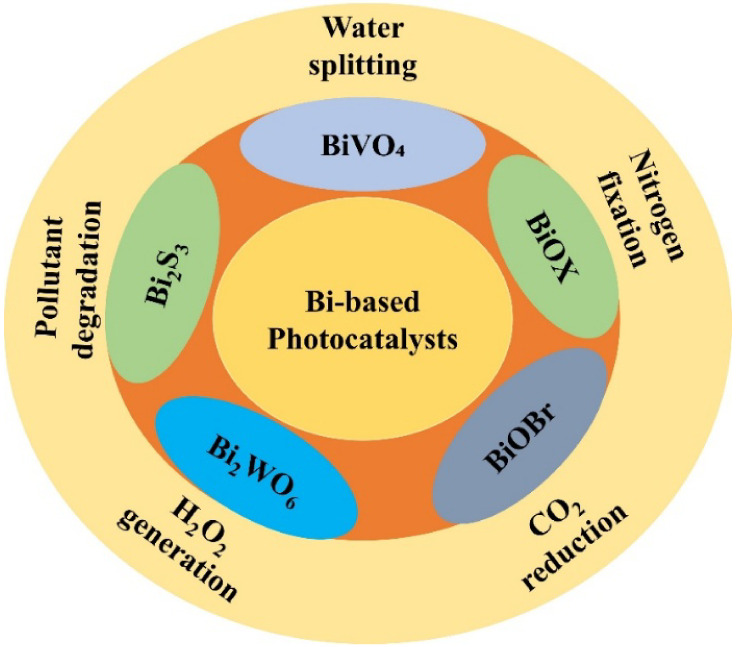
Schematic representation of Bi-based photocatalysts. The diagram highlights the key photocatalytic applications of Bi-based materials, including water splitting, pollutant degradation, H_2_O_2_ generation, CO_2_ reduction, and nitrogen fixation.

To overcome intrinsic limitations such as poor charge mobility or rapid electron–hole recombination, multiple material engineering approaches have been developed. Heterojunction engineering particularly the construction of type-II and Z-scheme systems has proven effective in enhancing charge separation and extending carrier lifetimes.^78^ Furthermore, doping with metals or nonmetal elements enables fine-tuning of the bandgap and fosters defect mediated charge migration pathways. Despite these advances, several challenges persist, including limited quantum efficiency and difficulties in scalable synthesis with controlled morphology and crystallinity. Future research must focus on in situ characterization techniques, computational modeling, and the development of robust synthesis protocols to unlock the full potential of bismuth-based photocatalysts in solar-driven chemical transformations and pollutant degradation.^79^

## 4 Photocatalytic Degradation of Antibiotics

### 4.1 Amoxicillin

Amoxicillin (AMX) is a broad-spectrum β-lactam antibiotic, similar to ampicillin, with enhanced oral absorption. It functions by irreversibly inhibiting penicillin-binding proteins (PBPs), which are essential enzymes in the final stages of bacterial cell wall peptidoglycan synthesis.^80^ AMX exhibits bactericidal activity against Gram-positive and Gram-negative bacteria and is widely used in human medicine and livestock production due to its broad-spectrum β-lactam structure.^81^

The influence of molecular characteristics on photocatalytic degradation can be described sequentially. First, the intrinsic molecular structure, including aromatic rings, conjugated π-systems, and heterocyclic frameworks, governs light absorption behavior, HOMO–LUMO energy distribution, and electron transfer potential, thereby affecting interaction with photoexcited catalysts.^82^ Second, functional groups such as −NH_2_, −OH, amide, and carboxyl moieties determine the distribution of electron density within the molecule. Electron-donating groups enhance susceptibility to oxidative attack by ROS, particularly •OH and h^+^, while electron-withdrawing groups modify oxidation potential and redirect degradation pathways.^83^ Third, structurally strained components, such as the β-lactam ring in AMX, act as preferential reactive centers where oxidative cleavage and ring-opening reactions are initiated. Fourth, the ionization state of the antibiotic, controlled by its p*K*_a_ values and environmental pH, dictates surface charge characteristics and adsorption affinity toward photocatalysts.^84^ Electrostatic attraction promotes surface-mediated oxidation via direct hole transfer, whereas electrostatic repulsion favors indirect oxidation in the bulk solution through diffusible ROS such as •OH and O_2_•^–^. Additionally, solution pH influences ROS speciation and redox potential, thereby altering degradation efficiency. Finally, adsorption orientation on the catalyst surface further determines which functional groups are exposed to oxidative species, mineralization efficiency, and the potential generation of toxic transformation products during advanced oxidation processes.^82^ Table 2 presents a summarized overview of AMX degradation through photocatalysis using different types of photocatalysts.

**Table 2:** Comparison of Amoxicillin Photodegradation under Diverse Experimental Conditions

S. No.	Antibiotic	Photocatlayst	Light	Concentration (mg/L^–1^)	Degradation time (min)	Degradation efficiency (%)	Reference
1.	Amoxicillin	TiO_2_@nZVI/PS	Visible light	20	60	99%	^93^
2.	Amoxicillin	C/Sn_2_ Ta_2_O_7_/SnO_2_	Simulated sunlight	20	120	88.3%	^94^
3.	Amoxicillin	TiO_2_–Cr	Visible light	10	90	100%	^95^
4.	Amoxicillin	Ag/TiO_2_ /mesoporous g-C_3_ N_4_	Visible light	5	60	_	^96^
5.	Amoxicillin	GO/TiO_2_	UV light	50	60	99%	^87^
6.	Amoxicillin	TiO_2_	UV light	10	150	80%	^97^
7.	Amoxicillin	MIL-53(Al)/ZnO	Visible light	10	60	100%	^98^
8.	Amoxicillin	BiVO	Visible light	5	90	97.45%	^99^
9.	Amoxicillin	CuI/FePO_4_	Visible light	10	90	90%	^100^
10.	Amoxicillin	Pd_1.00_/TiO_2_	Solar light	5	60	96.7%	^101^

Bergamonti et al. studied AMX degradation using TiO_2_-chitosan (TiO_2_/CS) scaffolds under UV–vis light. These scaffolds were fabricated via 3D printing by dispersing 6.0% w/v chitosan and 1.0% w/v commercial P25-TiO_2_. The resulting TiO_2_/CS system demonstrated excellent reusability and maintained high photocatalytic activity.^85^ Chinnaiyan et al. conducted a study on the removal of AMX from simulated hospital wastewater in a 200 mL photoreactor. The process involved TiO_2_ as the photocatalyst and UV light at a wavelength of 365 nm, provided by a 125 W mercury vapor lamp. Under optimal conditions-pH 7.6, a TiO_2_ concentration of 563 mg/L, and an initial AMX concentration of 10 mg/L the system achieved a maximum degradation efficiency of 90.0% after 150 min of irradiation.^86^ Balarak et al. studied the photocatalytic degradation of amoxicillin using TiO_2_ NPs supported on graphene oxide (GO/TiO_2_) under UV light. Nearly 100% degradation was achieved at pH 6 with 0.4 g/L of catalyst and 50 mg/L AMX using 36 W UV intensity. The catalyst showed excellent reusability over four cycles. Mineralization was confirmed by the presence of NH_4_^+^, NO_3_^–^, and SO_4_^2–^ ions.^87^ Wahyuni et al. developed Cu-doped TiO_2_ and applied it for AMX degradation under visible light. Results showed TiO_2_ performed better under UV light, while Cu-TiO_2_ was more effective under visible light.^88^ Qilong et al. designed a TiO_2_ based ceramic membrane photocatalytic reactor that efficiently degrades AMX across a wide pH range, showing stable performance and wastewater treatment.^89^ Al-Musawi et al. discussed the limitations of using TiO_2_ as a supercapacitor in environmental applications, despite its effectiveness. Its large-scale use is restricted due to three main issues: limited absorption of sunlight (3–5% due to UV-dependent activation, and nanoparticle agglomeration, which lowers its catalytic performance for AMX removal.^90^ Ellepola et al. studied TiO_2_ anatase for the photodegradation of AMX and found a 4.5-fold increase in degradation under light exposure. Nearly complete AMX degradation was achieved under UV–vis light within 300 h.^91^ Huang et al. prepared carbon-enriched g-C_3_N_4_ nanosheets (NSs) with high surface areas through a straightforward thermal polymerization technique. These NSs demonstrated excellent photocatalytic performance in degrading AMX (Figure 4 a–d). Additionally, the catalyst exhibited high stability and maintained its degradation efficiency across different media conditions, highlighting its strong potential for practical applications in AMX treatment.^92^

**Figure 4:**
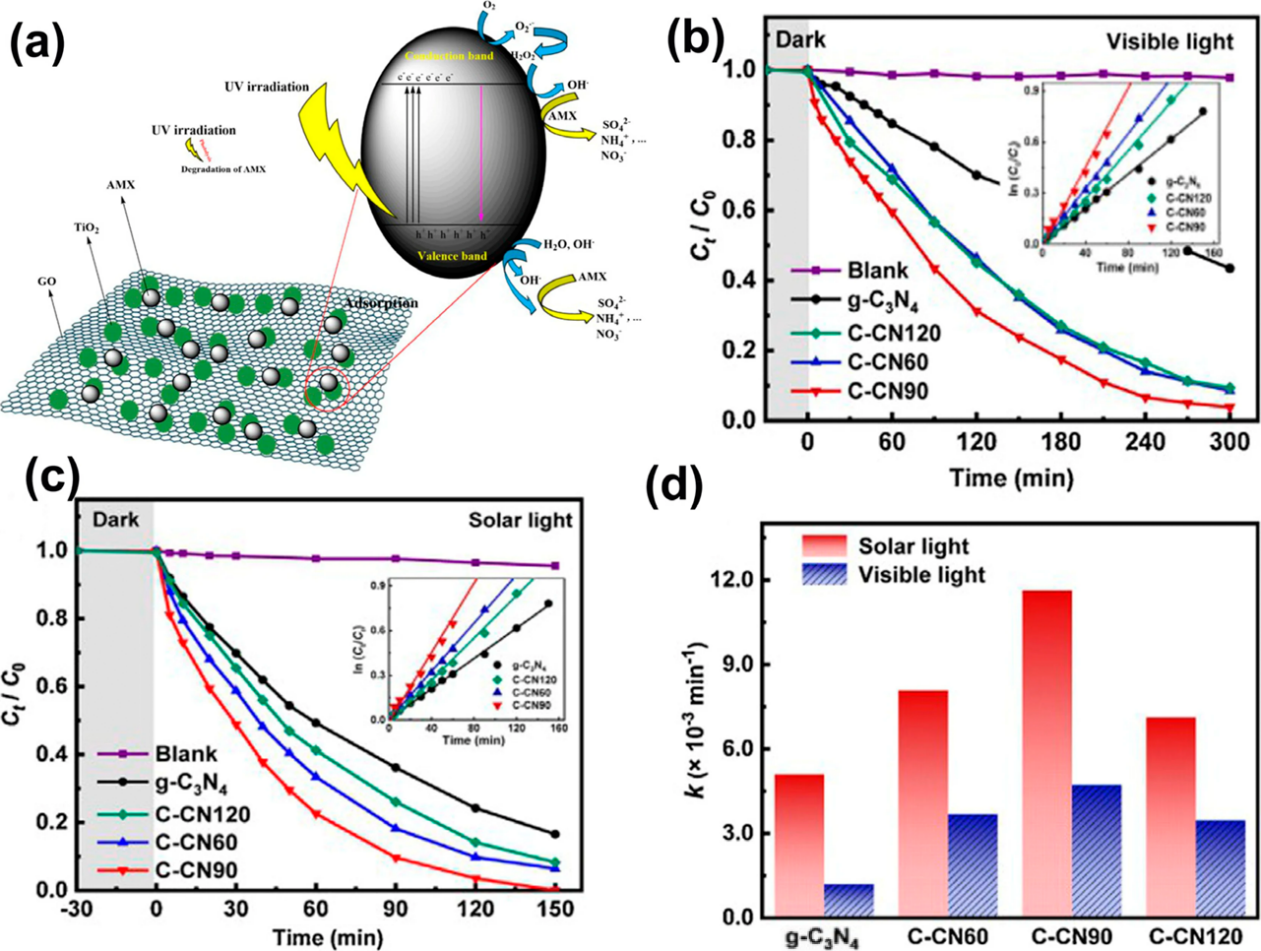
(a) The proposed mechanism of amoxicillin degradation on the GO/TiO_2_ surface, (b) visible light and (c) simulated solar light, (d) the degradation rate constants for both light sources. Reprinted with permission from Huang, D., Sun, X., Liu, Y., Ji, H., Liu, W., Wang, C. C., Ma, W., and Cai, Z. (2021). A carbon-rich g-C_3_N_4_ with promoted charge separation for highly efficient photocatalytic degradation of amoxicillin. *Chinese chemical letters*, *32*(9), 2787–2791. Copyright 2021 Elsevier.

### 4.2 Ciprofloxacin

Ciprofloxacin (CIP) is a second-generation fluoroquinolone antibiotic widely used in human and veterinary medicine due to its broad-spectrum activity against Gram-negative bacteria and certain Gram-positive strains.^102^ CIP exerts its antibacterial effect by inhibiting DNA gyrase and topoisomerase IV, enzymes essential for bacterial DNA replication and transcription. The molecule exhibits concentration-dependent bactericidal activity, and its physicochemical properties, including high solubility and amphoteric character, enable extensive tissue penetration and variable ionization states depending on environmental pH.^103^ These features confer chemical stability, enhanced electron density, and reactive sites, thereby influencing environmental interactions.^103^

In aqueous systems, CIP demonstrates strong binding potential with natural organic matter and surfaces due to its amphoteric nature, which affects both its mobility and susceptibility to degradation. The role of molecular and electronic features in photocatalytic degradation can be outlined sequentially. The conjugated quinolone core with delocalized π-electrons facilitates light absorption and promotes electron transfer processes between CIP and photoexcited catalysts.^104^ The functional groups, including ketone, fluorine, and piperazine moieties, modulate electron density, influencing susceptibility to ROS such as •OH, O_2_•^–^, and h^+^. The electron-withdrawing fluorine atom stabilizes the molecule but can redirect oxidative attack to other reactive centers.^105^

CIP’s amphoteric piperazine ring undergoes protonation or deprotonation depending on solution pH, thereby controlling electrostatic interactions with catalyst surfaces. Positively charged CIP species adsorb more readily onto negatively charged photocatalysts, promoting surface-mediated oxidation, whereas neutral or negatively charged forms are more exposed to ROS in the bulk solution.^102^ The presence of multiple reactive sites governs degradation pathways, including defluorination and hydroxylation, ultimately determining intermediate formation, mineralization efficiency, and the potential formation of toxic byproducts. Finally, adsorption orientation and site accessibility on catalyst surfaces further regulate ROS interactions and overall photocatalytic efficiency.^101^ Table 3 summarizes the CIP degradation using semiconductor-based photocatalytic degradation under different conditions such as light source, concentration and time.

**Table 3:** Photocatalytic Removal of Ciprofloxacin under Various Conditions

S. No.	Antibiotic	Photocatalyst	Light	Concentration (mg/L^–1^)	Degradation time (min)	Degradation efficiency (%)	Reference
1.	Ciprofloxacin	TiO_2_/N	UV light	30.0	120	87.87%	^109^
2.	Ciprofloxacin	NCuTiO_2_/CQD	Visible light	20.0	180	_	^110^
3.	Ciprofloxacin	Au-RGO/TiO_2_	Visible light	10	180	96.93%	^111^
4.	Ciprofloxacin	TiO_2_/Ce	UV light	40.0	180	90–93%	^112^
5.	Ciprofloxacin	Bi_2_O_2_/CO_3_	Visible light	10.0	60	76.8%	^113^
6.	Ciprofloxacin	Fe_3_O_4_/Bi_2_WO_6_	Visible light	10.0	25	99.7%	^114^
7.	Ciprofloxacin	ZnO–SnO_2_–Zn _2_SnO_4_	Stimulate sunight	10.0	95	_	^115^
8.	Ciprofloxacin	TiO_2_/WO_3_	UV light	20.0	120	100%	^116^
9.	Ciprofloxacin	CeO_2_/ZnO	UV light	10.0	60	60%	^117^
10.	Ciprofloxacin	rGO/Bi_4_O_5_Br_2_	Visible light	10.0	60	97.6%	^118^

Various studies have highlighted the use of modified photocatalysts to enhance their photocatalytic performance.^106^ A common approach adopted by researchers involves doping the photocatalysts with transition metals to improve their efficiency. Şimşek et al. prepared boron-doped TiO_2_ using a solvothermal approach to break down CIP under visible light. Doping levels (2–8%) enhanced degradation, with 8% B/TiO_2_ achieving the highest efficiency of 88.32%. Optimal conditions included 1.1 g/L catalyst, pH 7.1, and 7.23 mM H_2_O_2_. The doped material exhibited a reduced bandgap, improved photocatalytic performance, and good reusability over five cycles.^106^ Malakootian et al. developed CeO_2_Ag/AgBr photocatalyst by in situ depositing AgBr on CeO_2_. This material showed significantly improved photocatalytic performance for CIP degradation. The enhancement was attributed to faster charge transfer and better separation of photogenerated electron–hole pairs.^107^ Pattnaik et al. reported a green, aqueous bithermal method to synthesize exfoliated g-C_3_N_4_ NPs, which were characterized using XRD, UV-DRS, FESEM, PL, and TEM. Their photocatalytic performance was evaluated by degrading CIP under solar irradiation. Compared to bulk g-C_3_N_4_, the exfoliated material showed significantly enhanced activity, attributed to its ∼3-fold higher surface area and ∼2.5-times greater photocatalytic efficiency. Under optimal conditions, 1 g L^–1^ exfoliated g-C_3_N_4_ degraded 78% of a 20 ppm of CIP solution within 1 h of sunlight exposure. Scavenger experiments helped identify the active species involved, demonstrating the potential of this scalable, green exfoliation approach (Figure 5).^108^

**Figure 5:**
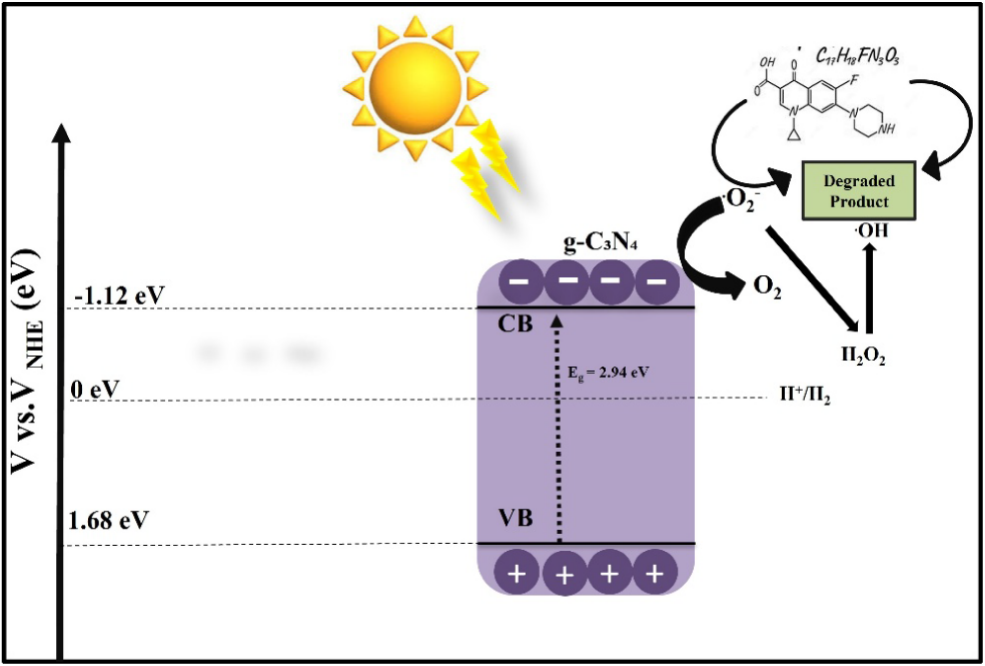
Ciprofloxacin degradation mechanism using a g-C_3_N_4_ photocatalyst.

### 4.3 Sulfamethoxazole

Sulfamethoxazole (SMX) is a widely used sulfonamide antibiotic that inhibits bacterial folic acid synthesis by competitively binding to dihydropteroate synthase. It exhibits broad-spectrum activity against both Gram-positive and Gram-negative bacteria and is commonly administered alone or in combination with trimethoprim for the treatment of urinary tract infections, respiratory infections, and certain gastrointestinal infections.^119^ SMX has high water solubility, moderate lipophilicity, and a weakly acidic character due to its sulfonamide −NH–SO_2_– functional group. Structurally, it contains a benzene ring substituted with an amino group (−NH_2_) at the para position, an isoxazole heterocycle, and the sulfonamide linkage, which together define its chemical stability, electron distribution, and reactivity in aqueous environments. Its amphoteric nature allows pH-dependent ionization, which strongly affects adsorption onto surfaces and interactions with reactive species.^120^

The photocatalytic degradation of SMX is strongly influenced by its molecular and electronic characteristics. The conjugated aromatic ring and isoxazole moiety facilitate light absorption and electron transfer with photoexcited catalysts, while functional groups such as the amino and sulfonamide moieties create localized electron-rich and electron-deficient sites that guide reactivity toward ROS, including •OH, O_2_•^–^, and h^+^.^119^ Electron-donating groups enhance susceptibility to oxidative attack, whereas electron-withdrawing groups can stabilize certain parts of the molecule and redirect ROS attack to other reactive centers. The ionization state of SMX, controlled by solution pH relative to its p*K*_a_ values, influences its surface charge and adsorption affinity on photocatalysts. Positively or neutrally charged species adsorb effectively, promoting surface-mediated oxidation, whereas negatively charged species are more exposed to bulk ROS-mediated degradation.^120^ Multiple reactive centers, including the aromatic ring, and sulfonamide linkage, determine preferential sites for ROS attack and dictate pathways such as hydroxylation, sulfonamide cleavage, and ring-opening reactions. Additionally, the orientation and accessibility of SMX on catalyst surfaces regulate which functional groups interact with ROS, ultimately influencing degradation kinetics, mineralization efficiency, and the potential generation of toxic byproducts.^120^ Table 4 highlights the performance of semiconductor photocatalysts in the efficient breakdown of SMX.

**Table 4:** Sulfamethoxazole Removal by Different Photocatalysts under Varying Conditions

S. No.	Antibiotic	Photocatlayst	Light	Concentration (mg/L^–1^)	Degradation time (min)	Degradation efficiency (%)	Reference
1.	Sulfamethoxazole	P-TiO_2_/g-C_3_N_4_	Visible light	10.0	90	87%	^18^
2.	Sulfamethoxazole	MoS_2_@CoS_2_	Visible light	20.0	180	95%	^125^
3.	Sulfamethoxazole	CoCuS@TiO_2_	Solar light	5.0	120	100%	^126^
4.	Sulfamethoxazole	NSFe-TiO_2_	UV light	20.0	120	90%	^127^
5.	Sulfamethoxazole	CuxO/TiO_2_	Visible light	10	60	98.2%	^128^
6.	Sulfamethoxazole	N–TiO_2_@C	Visiblel light	20.0	140	99%	^129^
7.	Sulfamethoxazole	TiO_2_/BC	UV light	30.0	60	89%	^130^
8.	Sulfamethoxazole	Bi_2_WO_6_/RGO	Simulated sunlight	10.0	480	70.9%	^131^
9.	Sulfamethoxazole	ZnO/ZnIn_2_S_4_	Visible light	2.5	390	74.9%	^132^
10.	Sulfamethoxazole	Bi_2_O_3_–TiO_2_/PAC	Visible light	20.0	120	67.9%	^133^

Drzymała and Kalka observed that toxicity generally increases with the complexity of the solution before treatment. After 6 min of ozonation, the SMX sample exhibited reduced toxicity for all tested organisms except *Lemna minor*. This suggests selective toxicity changes depending on the organism and treatment time.^121^ Jesus et al. assessed the toxicity of SMX, methylparaben, paracetamol, and carbamazepine both individually and in mixtures containing various drug combinations before and after ozonation. Their study aimed to understand how ozonation affects the toxicity of these pharmaceuticals in different contexts.^122^ Gong et al. examined the UV photodegradation of SMX using a reusable CoFe_2_O_4_/TiO_2_ catalyst. SMX was fully degraded within 5 h, along with about 50% mineralization, and the treated solution showed much lower toxicity to *Chlorella vulgaris* and *Artemia salina*. Mechanistic analysis based on LC-ESI-MS identified 16 transformation products, including four previously unreported intermediates. The degradation followed four main routes: hydroxyl radical-driven oxidation, S–N bond cleavage, amino group modification, and structural rearrangement. Hydroxylation was the predominant pathway, leading to ring oxidation, bond breakage, and formation of smaller, less harmful compounds, explaining the reduced toxicity after treatment (Figure 6).^123^

**Figure 6:**
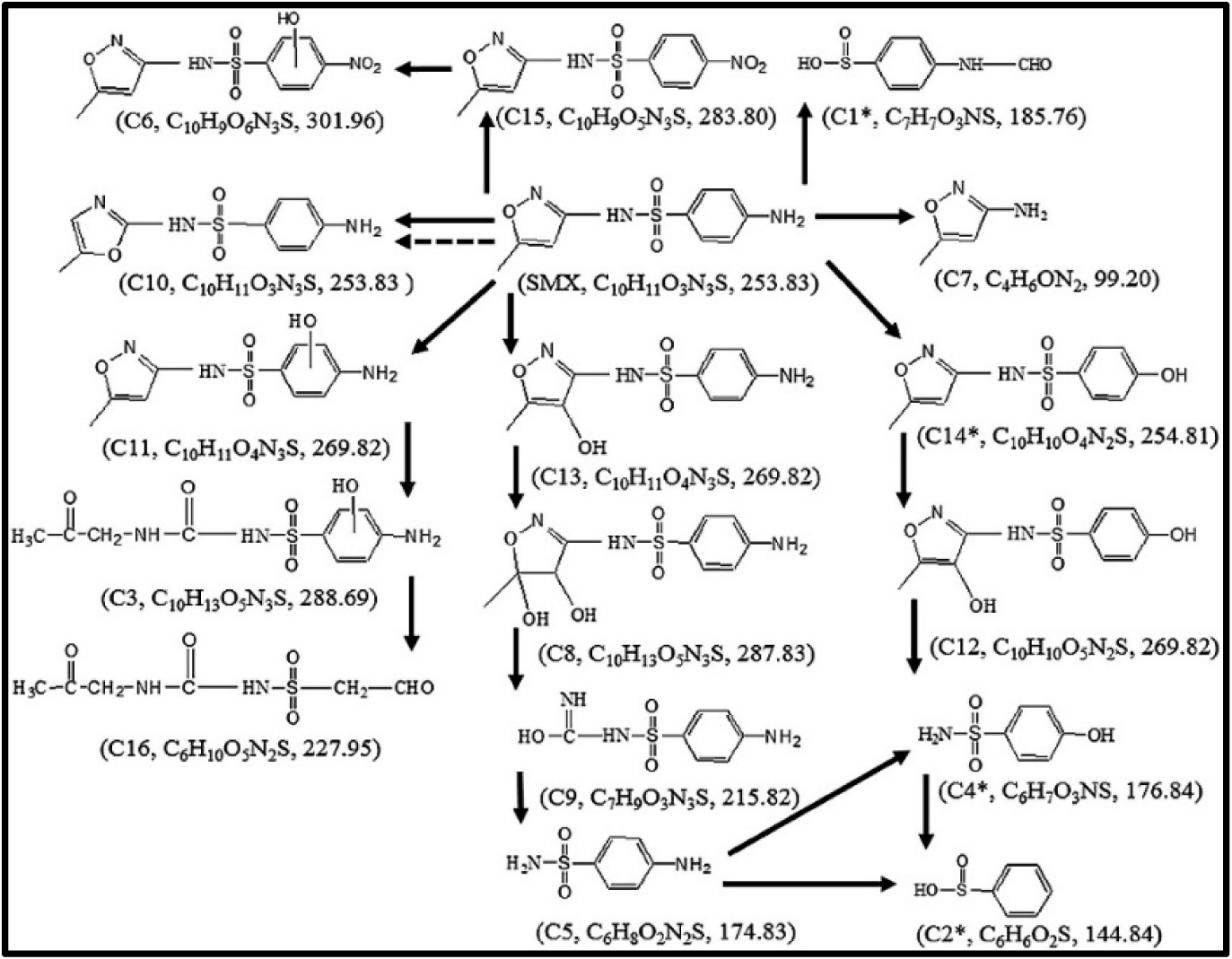
Mechanism of photochemical degradation of SMX. Reprinted with permission from Gong, H., and Chu, W. (2016). Determination and toxicity evaluation of the generated products in sulfamethoxazole degradation by UV/CoFe_2_O_4_/TiO_2_. *Journal of hazardous materials*, 314, 197–203. Copyright 2016 Elsevier.

Li et al. investigated the influence of various adsorption sites on biochar derived from corncobs carbonized at 800 °C (CC800) on the uptake of SMX. Their results revealed a three-step decrease in adsorption across a pH range of 2–10, reflecting different adsorption mechanisms at distinct sites. Optimal adsorption occurred at pH 2, 25 °C, in the dark, with 0.1 g adsorbent, achieving a maximum capacity of 425 mg/g. Figure 7 illustrates the proposed SMX adsorption mechanism on CC800 and the associated adsorption energies.^124^

**Figure 7:**
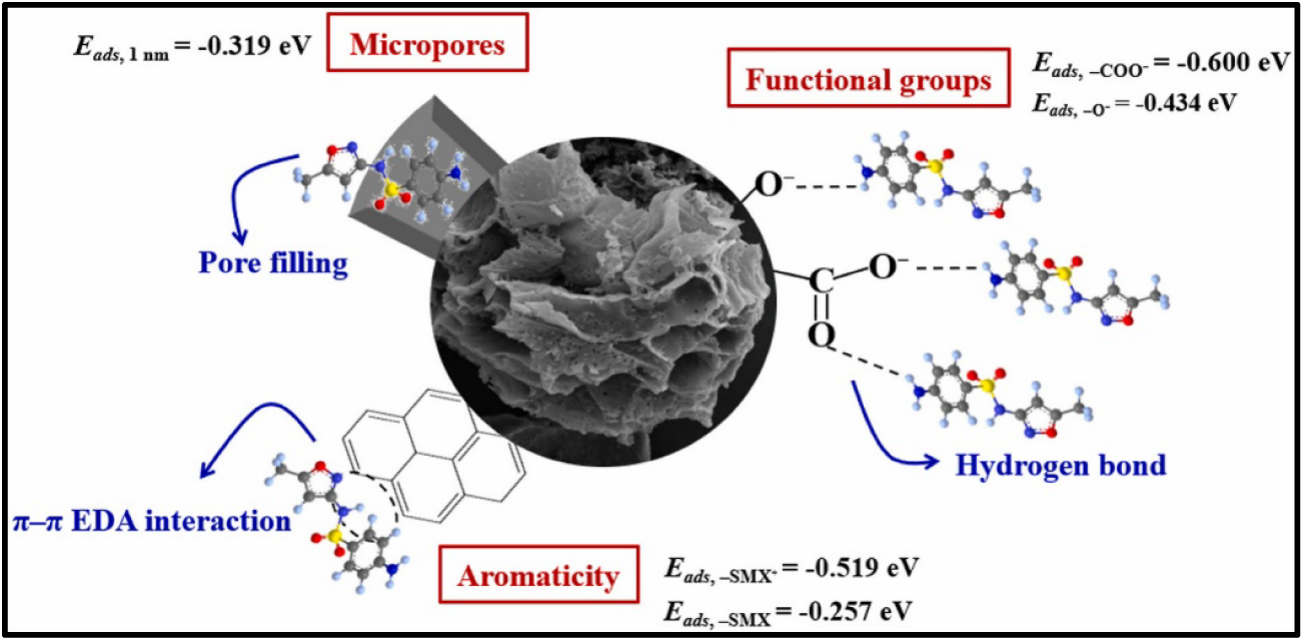
Mechanism of SMX adsorption on biochar. (Reprinted with permission from Li, Y., Wang, B., Shang, H., Cao, Y., Yang, C., Hu, W., Feng, Y., and Yu, Y. (2023). Influence of adsorption sites of biochar on its adsorption performance for sulfamethoxazole. *Chemosphere*, 326, 138408. Copyright 2023 Elsevier.

### 4.4 Tetracycline

Tetracycline (TC) is a broad-spectrum antibiotic that inhibits bacterial protein synthesis by binding to the 30S ribosomal subunit, thereby preventing the attachment of aminoacyl-tRNA. It is active against a wide range of Gram-positive and Gram-negative bacteria and is commonly used in human and veterinary medicine to treat respiratory, urinary, and gastrointestinal infections.^134^ Structurally, TC contains a tetracyclic naphthacene ring system with multiple hydroxyl (−OH), keto (C=O), amide (−CONH−), and amino (−NH_2_) functional groups, which confer significant chemical reactivity, electron delocalization, and the ability to chelate metal ions.^135^ These features also allow TC to exist in different ionization states depending on the environmental pH, making it amphoteric and enabling cationic, zwitterionic, or anionic forms. This ionization behavior strongly influences its solubility, adsorption onto catalyst surfaces, and interaction with ROS.^136^ The photocatalytic degradation of TC is largely determined by its molecular and electronic characteristics. The conjugated tetracyclic core facilitates light absorption and promotes electron transfer between TC and photoexcited catalysts. Functional groups, amino, keto, and amide moieties, create electron-rich and electron-deficient sites that govern the reactivity toward ROS, such as hydroxyl radicals •OH, O_2_•^–^, and h^+^.^136^ Electron-donating groups increase the susceptibility of specific molecular regions to oxidative attack, whereas electron-withdrawing groups stabilize certain portions and direct ROS attack toward other reactive centers. The ionization state of TC, which varies with pH and the surrounding environment, determines electrostatic interactions with photocatalyst surfaces. Positively charged or neutral forms exhibit stronger adsorption to negatively charged surfaces, enhancing direct surface-mediated oxidation, while negatively charged species are more exposed to bulk ROS-mediated degradation in solution.^137^ The multiple reactive centers present in TC, including the tetracyclic rings and various functional groups, guide preferential ROS attack and dictate degradation pathways, such as hydroxylation, deamination, keto group oxidation, and complex rearrangements.^136^ Additionally, the spatial orientation of TC molecules on catalyst surfaces influences which functional groups are accessible to ROS, ultimately regulating degradation kinetics, mineralization efficiency, and the potential generation of toxic transformation products.^137^ Table 5 summarizes the performance of various semiconductor technologies in TC degradation.

**Table 5:** Photocatalytic Efficiency for Tetracycline Degradation under Varying Conditions

S. No.	Antibiotic	Photocatalyst	Light	Concentration (mg/L^–1^)	Degradation time (min)	Degradation efficiency (%)	Reference
1.	Tetracycline	CQDs@TNT	Visible light	0.001	80	100%	^126^
2.	Tetracycline	Ag_3_VO_4_/WO_3_	Visible light	10	30	71.2%	^144^
3.	Tetracycline	SnO_2_/g-C_3_N_4_	Visible light	30	120	95.9%	^145^
4.	Tetracycline	Pb/MoO_4_	Simulated light	20	120	50%	^146^
5.	Tetracycline	TiO_2_/g-C_3_N_4_	Simulated sunlight	20	100	89%	^147^
6.	Tetracycline	Ag/Ag_2_S/Bi_2_ MoO_6_	Visible light	20	120	87.3%	^148^
7.	Tetracycline	C-doped C_3_N_4_/Bi_12_O_17_C_l2_	Visible light	20	60	_	^149^
8.	Tetracycline	Cu_2_O–TiO_2_	Visible light	100	60	100%	^150^
9.	Tetracycline	MoS_2_/TiO_2_	Visible light	10	100	84%	^151^
10.	Tetracycline	Ti_3_C_2_@TiO_2_	Visible light	20	90	90%	^152^

Tri et al. evaluated Ag-doped g-C_3_N_4_ for TC degradation under solar light, achieving a 96.8% degradation rate with 3 mmol Ag after 2 h. When tested on hospital wastewater, the material showed 89.6% efficiency. The photocatalyst remained stable and effective after six reuse cycles. Its strong performance is credited to improved charge separation and transfer.^138^ Jodeyri et al. created an Ag/C_3_N_4_ clinoptilolite nanocomposite for degrading TC antibiotics under simulated solar light. The nanophotocatalyst achieved 90% degradation within 3 h, outperforming Ag/C_3_N_4_, C_3_N_4_-clinoptilolite, and C_3_N_4_ alone. This improved efficiency is linked to clinoptilolite’s increased surface area and the surface plasmon resonance (SPR) effect of silver.^139^ Xue et al. explored the use of plasmonic Au nanomaterials in enhancing photocatalysis. Their Au/Pt/g-C_3_N_4_ photocatalyst exhibited a degradation rate 3.4 times higher than pure g-C_3_N_4_ for TC hydrochloride under visible light. The improvement was mainly due to gold’s plasmonic effect, which broadened light absorption, along with platinum’s role as an electron sink, boosting overall activity.^140^ Wang et al. prepared Bi_4_Ti_3_O_12_ NSs using the molten salt method, optimizing the ratio of {001} top facets to {010}/{100} lateral facets. This configuration showed the highest photocatalytic efficiency for degrading TC-HCl. The degradation primarily involved photogenerated holes (h^+^), while superoxide radicals (O_2_^–^) had a minor effect.^141^ Rawat et al. synthesized carbon quantum dots (CQDs) and decorated TiO_2_ nanotubes (CQDs@TNT), which reduced the band gap by 2.92 eV and showed quantum confinement effects. Characterization confirmed structural changes and elemental composition. The CQDs@TNT exhibited excellent photocatalytic degradation of TC, following pseudo second-order kinetics.^142^ Chen et al. prepared an AgI/BiVO_4_ heterostructured photocatalyst using in situ precipitation. Under visible light, it degraded TC with 94.91% efficiency in 60 min. This was higher than BiVO_4_ (62.68%) and AgI (75.43%) under the same conditions (Figure 8a and b).^143^

**Figure 8:**
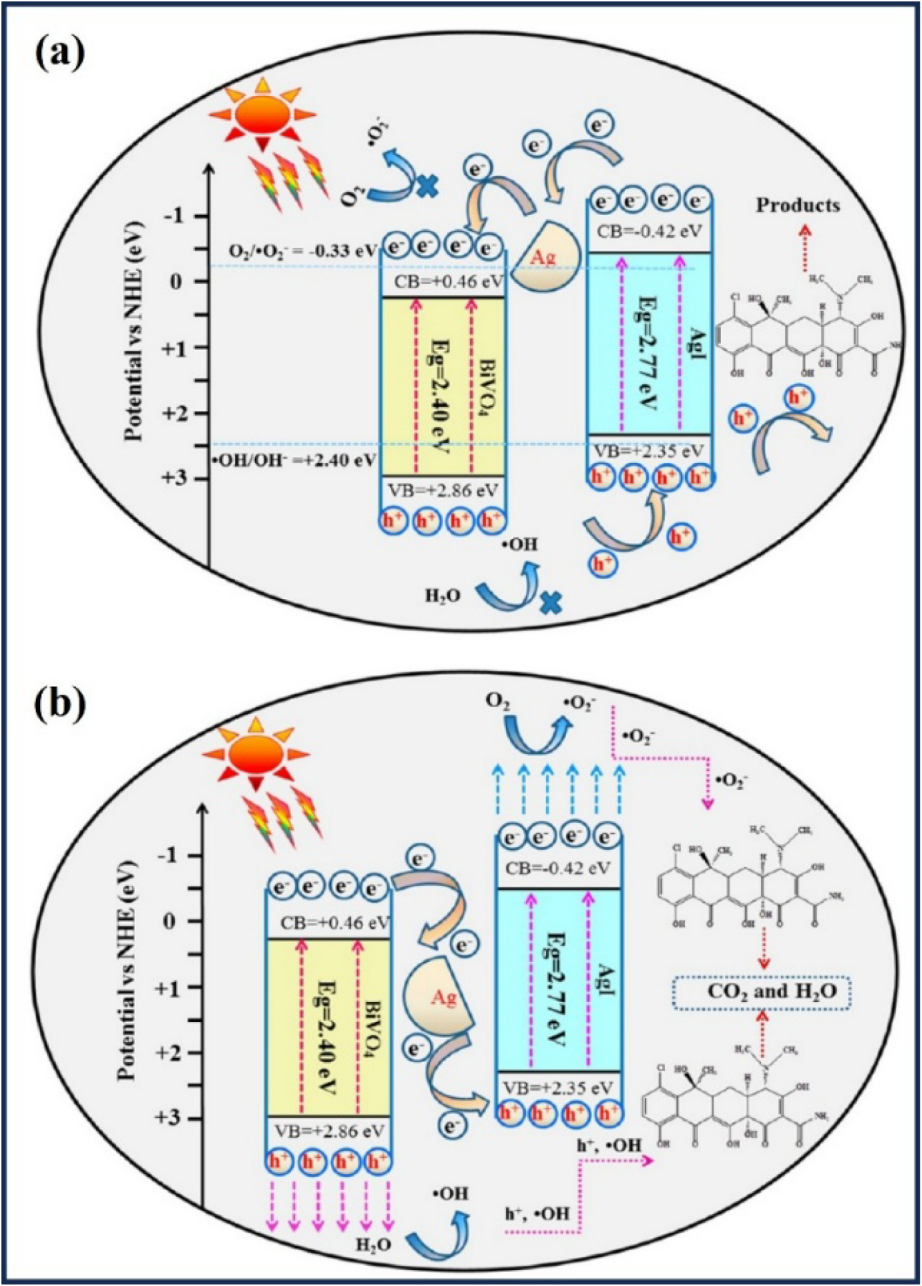
Mechanism for tetracycline degradation under visible light using the AgI/BiVO_4_ nanocomposite: (a) Conventional mechanism, and (b) Z-scheme heterojunction. Reprinted with permission from Chen, F., Yang, Q., Sun, J., Yao, F., Wang, S., Wang, Y., Wang, X., Li, X., Niu, C., Wang, D., and Zeng, G. (2016). Enhanced photocatalytic degradation of tetracycline by AgI/BiVO_4_ heterojunction under visible-light irradiation: mineralization efficiency and mechanism. *ACS applied materials and interfaces*, 8(48), 32887–32900. Copyright 2016 American Chemical Society.

## 5 Challenges and Future Perspectives

Despite promising laboratory-scale results, the practical application of 1D nanostructured photocatalysts for antibiotic degradation in wastewater faces several critical challenges that must be overcome before industrial deployment. A major short-term limitation is the scalable and cost-effective synthesis of 1D photocatalysts. Conventional fabrication methods, including hydrothermal growth, sol–gel processing, and electrospinning, are effective at laboratory scale but are energy-intensive, and costly. To enable industrial production, future research should focus on low-temperature solution processing, continuous-flow synthesis, template-free growth, and green chemistry approaches using abundant precursors and recyclable solvents, while preserving crystallinity, and catalytic activity. Another immediate challenge is the narrow light absorption range of conventional 1D photocatalysts, which restricts their efficiency under solar irradiation. Although doping, heterojunction formation, and coupling with carbon-based materials can enhance visible-light response, these modifications may compromise long-term stability or introduce environmental risks. Materials development should aim for broadband solar absorption across 400–700 nm and apparent quantum efficiency exceeding 10% under visible light, guided by computational band-structure design and the use of nontoxic dopants and stable cocatalysts. In the midterm, reusability and recovery remain significant bottlenecks. Suspended photocatalysts are difficult to separate from treated water, while immobilization on substrates often reduces active surface area and catalytic efficiency. Repeated use can lead to photocorrosion, aggregation, or surface fouling, causing performance loss. Scalable immobilization strategies, such as coating on ceramic monoliths, stainless-steel meshes, or polymer membranes, must be developed with minimal mass-transfer limitations, ensuring retention of at least 85–90% of initial degradation efficiency over multiple cycles. Incomplete mineralization of antibiotics is another critical concern, as partial degradation can produce potentially toxic intermediates. Long-term priorities focus on multifunctional applications, including simultaneous antibiotic degradation and sustainable hydrogen production via photocatalytic water splitting. The high surface area, directional charge transport, and tunable band structures of 1D nanostructures make them promising candidates for visible-light-driven hydrogen evolution. Future systems should target hydrogen evolution rates of 100–500 μmol h^–1^ g^–1^ while maintaining high antibiotic degradation efficiency. Achieving this dual functionality requires precise optimization of photocatalyst composition, and reactor design to allow simultaneous pollutant removal and gas collection. Industrial implementation will also require coordinated efforts in material synthesis, reactor engineering, and techno-economic analysis, with clear pilot-scale performance benchmarks to demonstrate scalability, energy efficiency, and environmental sustainability.

## 6 Conclusion

The growing concern over antibiotic contamination in aquatic systems calls for innovative and efficient treatment strategies beyond conventional methods. 1D nanostructured photocatalysts have emerged as advanced materials capable of promoting the oxidative degradation of antibiotics through enhanced light absorption and increased surface reactivity. Although laboratory studies demonstrate significant performance advantages, key limitations including synthesis scalability, and incomplete understanding of degradation by products must be addressed before field-scale implementation can be realized. This review provides a distinct contribution by systematically correlating the intrinsic properties of 1D nanostructured photocatalysts with their reproducibility, long-term stability, and reusability in antibiotic degradation. By integrating synthesis strategies, photocatalytic mechanisms, and performance consistency across repeated cycles, it advances current understanding toward realistic and sustainable water treatment applications.

Recent advances demonstrate that improved charge transport pathways and higher degradation efficiencies under visible-light irradiation clearly distinguish 1D nanostructured photocatalysts from conventional systems. Equally important is their ability to maintain catalytic activity over multiple reaction cycles, highlighting operational stability, resistance to photocorrosion, and structural durability as essential attributes for long-term application. Importantly, this review establishes a comparative framework linking antibiotic molecular structure with the rational selection of photocatalytic materials. Antibiotics containing complex aromatic rings and electron-rich functional groups, such as fluoroquinolones and tetracyclines, generally benefit from visible-light-responsive 1D heterostructures or carbon-coupled systems that enhance π–π interactions and charge transfer efficiency. In contrast, antibiotics with simpler β-lactam or sulfonamide structures are often effectively degraded by UV-active metal oxide nanorods or nanowires, where strong oxidative radical generation dominates the degradation pathway.

Therefore, material selection should not rely solely on morphology but rather on matching band structure, reactive species generation capability, and surface chemistry with the functional groups of the target antibiotic. By comparing degradation trends across antibiotic classes, this review emphasizes that tailored photocatalyst design based on antibiotic structure, light source, and operational conditions offers a more reliable strategy than a universal material approach. Future progress will depend on cost-effective fabrication techniques, comprehensive environmental risk assessments, and greater emphasis on synthesis reproducibility and performance consistency. From a sustainability perspective, the recoverability, reusability, and prolonged service life of 1D nanomaterials support reduced material consumption and secondary waste generation, aligning photocatalytic water treatment with circular economy principles. Collectively, these considerations provide a focused framework for advancing next-generation photocatalytic technologies aimed at the sustainable mitigation of antibiotic pollution in aquatic environments.
